# Safety of edoxaban for delayed bleeding in gastrointestinal endoscopic procedures with a high risk of bleeding

**DOI:** 10.1002/deo2.70018

**Published:** 2024-10-03

**Authors:** Ken‐ichi Mizuno, Junji Yokoyama, Osamu Shibata, Yuichi Kojima, Yuzo Kawata, Kazuya Takahashi, Kentaro Tominaga, Ikarasi Satoshi, Hayashi Kazunao, Shuji Terai

**Affiliations:** ^1^ Department of Endoscopy Niigata University Medical and Dental Hospital Niigata Japan; ^2^ Department of Gastroenterology Saiseikai Niigata Hospital Niigata Japan; ^3^ Division of Gastroenterology and Hepatology, Graduate School of Medical and Dental Science Niigata University Niigata Japan

**Keywords:** delayed bleeding, DOACs, edoxaban, EMR, ESD

## Abstract

**Objectives:**

There are limited reports on the safety of gastrointestinal endoscopic procedures in individuals taking edoxaban, one of the direct oral anticoagulants. We clarified the incidence of delayed bleeding in patients who were on edoxaban in the perioperative period of gastrointestinal endoscopic procedures with a high risk of bleeding.

**Methods:**

This was an investigator‐initiated, single‐center, open‐label, prospective, single‐arm study. Patients on warfarin or edoxaban undergoing endoscopy with a high risk of bleeding were enrolled from June 2018 to September 2021. Warfarin was replaced with edoxaban in patients on warfarin. Patients taking other direct oral anticoagulants, and antiplatelet drugs, were excluded. The primary endpoint was severe delayed bleeding (Common Terminology Criteria for Adverse Events [CTCAE] grades III–V) and the secondary endpoints included thromboembolism, all adverse events, any delayed bleeding (CTCAE grades I or II), and hospital stay durations.

**Results:**

Twenty‐one patients on edoxaban underwent high‐risk endoscopy. Three cases (14%) experienced CTCAE grade III delayed bleeding, requiring endoscopic hemostasis. No CTCAE grade I‐II delayed bleeding or thromboembolic events occurred. Cholangitis and aspiration pneumonia (conservatively treated) occurred during the hospital stay. The median length of hospital stay was 8 days (range 3—24 days). Patients with delayed bleeding had higher systolic blood pressure at admission and longer hospital stays.

**Conclusions:**

The delayed bleeding incidence in high‐risk endoscopic procedures for patients on edoxaban was acceptable. Higher blood pressure may be associated with increased risk, but further research is needed.

## INTRODUCTION

With the advancement of an aging society, the proportion of people receiving antithrombotic therapy to prevent cardiovascular and cerebrovascular disease is increasing. These include anticoagulants and antiplatelet agents.^1^, ^2^, ^3^ With the increasing use of gastrointestinal endoscopic procedures, such as endoscopic mucosal resection (EMR) and endoscopic submucosal dissection (ESD), gastrointestinal perforation and delayed bleeding are attracting attention as adverse events.^4^, ^5^, ^6^ Antithrombotic therapy is a risk factor for delayed bleeding after ESD.^7^, ^8^

Patients at low risk for thrombosis can usually discontinue antithrombotic agents, and appropriate discontinuation is reported to not increase the delayed bleeding rate.^9^, ^10^ Meanwhile, in high‐risk thrombosis groups, heparin bridging therapy (HBT) has been used to prevent thrombosis due to warfarin discontinuation, but there are reports that HBT actually increases the risk of delayed bleeding after ESD.^9^, ^11^

In recent years, direct oral anticoagulants (DOACs) have been used as a new anticoagulant for non‐valvular atrial fibrillation. They include the direct thrombin inhibitor dabigatran and the direct factor Xa inhibitors such as rivaroxaban, apixaban, and edoxaban.^12^, ^13^, ^14^, ^15^ There have been several reports regarding the influence and management of DOACs in endoscopic procedures, mainly in ESD.^11^, ^16^, ^17^, ^18^, ^19^, ^20^, ^21^, ^22^, ^23^, ^24^, ^25^, ^26^, ^27^ The rate of delayed bleeding due to gastric ESD with DOACs was higher but not different from that of warfarin. Furthermore, when combined with HBT, the delayed bleeding rate of DOACs and warfarin increased.^18^ It has also been reported that DOACs increase the rate of delayed bleeding in ESD of the esophagus and colorectum.^19^, ^21^, ^22^ There are reports that there are differences in the delayed bleeding rates between DOACs, but no consensus has been reached in that regard.^11^, ^18^ Edoxaban is administered once daily, and its dosage can be adjusted based on factors such as body weight, renal function, and bleeding risk. Therefore, the number of patients taking edoxaban is expected to increase in gastrointestinal neoplastic diseases, which often occur in the elderly. Edoxaban has been reported to have fewer fatal bleeding adverse events than other DOACs when used for the prevention and treatment of stroke in patients with atrial fibrillation.^28^, ^29^ However, there are few reports on delayed bleeding in gastrointestinal endoscopic procedures that are limited to edoxaban, and these are only retrospective studies.^18^

In the present study, we clarified the incidence of delayed bleeding and thromboembolism, and the duration of hospital stays, of patients on edoxaban anticoagulation therapy in the perioperative period of gastrointestinal endoscopic procedures with a high risk of bleeding.

## METHODS

### Patients

This was an investigator‐initiated, single‐center, open‐label, prospective, single‐arm study.

We included consecutive patients who were on anticoagulant therapy with warfarin or edoxaban, for prevention of ischemic stroke and systemic embolism in non‐valvular atrial fibrillation or recurrence of venous thromboembolism, who underwent gastrointestinal endoscopic procedures with a high risk of bleeding from June 2018 to September 2021. Eligible patients were 20 years or older, had an Eastern Cooperative Oncology Group (ECOG) performance status of 0, 1, or 2, and had protocol‐defined adequate renal and hepatic function. Patients were excluded if they were on other DOACs, or antiplatelet agents such as aspirin, ticlopidine, clopidogrel, and cilostazol, in addition to warfarin or edoxaban..

There were approximately 45 endoscopy procedures with a high risk of bleeding with patients on anticoagulants annually at our institution. The ratio of warfarin to DOAC use was approximately 2:1, with edoxaban accounting for approximately 20% of DOAC cases. Therefore, there were approximately 30 patients on warfarin requiring heparin replacement, and about three patients on edoxaban, annually. We therefore estimated that our institution could accumulate 20 cases annually, with a total of 40 cases over a registration period of 2 years.

In two previous reports, the incidence of delayed bleeding was reported to be 20%–38%.^9^, ^30^ Assuming that 40 cases undergo endoscopy, the accuracy of the incidence of delayed bleeding, with a rate of 20% (eight cases), has a 95% confidence interval precision of ±12.4%, which we considered evaluable.

### Endoscopic procedures

The endoscopy procedures at high risk of bleeding performed in this study were determined according to the Japan Gastroenterological Endoscopy Society guidelines.^31^, ^32^ Although Per‐Oral Endoscopic Myotomy (POEM) is not listed in the Japan Gastroenterological Endoscopy Society guidelines, it was treated as an endoscopic procedure at high risk of bleeding in this study. Even when multiple lesions were resected by EMR or ESD, it was treated as a single operation.

ESD, EMR, and polypectomy in this study were performed based on standard procedures at our institution. ESD was performed using a Hook Knife (KD‐625LR or KD‐625QR; Olympus) or a Dual Knife (KD‐655L or KD‐655Q; Olympus), GIF‐Q260J or CF‐Q260JI (Olympus), and a high‐frequency generator (VIO300D; ERBE). A Coagrasper (FD‐410LR or FD‐411QR; Olympus) was used for blood vessel cauterization during or after ESD. EMR was performed based on the conventional EMR method, using submucosal injections and a snare with a loop size of 13 mm (Captivator Small Hex; Boston Scientific) or 20 mm (Rotatable Snare; Boston Scientific). Prophylactic clipping after polyp removal was routinely performed. POEM was performed under general anesthesia, and a Triangle Knife (KD‐645L; Olympus) was used for the creation of the submucosal tunnel and myotomy.

A scheduled second‐look endoscopy was performed on day 2 for patients who underwent ESD or EMR of gastric lesions or POEM.

A proton pump inhibitor (lansoprazole 30 mg) was intravenously injected twice a day before resumption of oral food intake. Once oral intake became possible, the acid inhibitor was switched to oral administration of esomeprazole (20 mg) or vonoprazan (20 mg) after ESD for upper GI lesions. All patients with gastric and duodenal lesions, resected by ESD or EMR, received acid inhibitors for more than 28 days post‐procedure.

### Management of anticoagulants

Patients on warfarin were switched to edoxaban for anticoagulant therapy after consultation with the attending physician. The dosage of edoxaban was adjusted based on body weight, renal function, and the presence of P‐glycoprotein inhibitors. Administration of edoxaban was continued until the day before the procedure, and discontinued on the morning of the procedure day. Administration of edoxaban was resumed after confirming the absence of clinical symptoms of gastrointestinal bleeding on the next day or the day after the treatment. If an endoscopy was performed the next day to check for bleeding, edoxaban was resumed after the examination. Edoxaban was continued during the 2‐month observation period.

### Adverse events

Adverse events were determined according to the Common Terminology Criteria for Adverse Events version 5.0 (CTCAE v5.0). The primary endpoint was the incidence of grade III to V delayed bleeding according to CTCAE v5.0 (e.g., hemorrhage requiring emergency endoscopic hemostasis or transfusion [grade III], life‐threatening/disabling hemorrhage [grade IV], and death [grade V]). Preventive hemostasis of exposed vessels over mucosal defects that did not meet the clinical criteria of bleeding at next‐day endoscopy was not included. The secondary endpoints included the incidence of thromboembolism, the incidence of all adverse events, the incidence rate of delayed bleeding of CTCAE v5.0 Grade I or II, and the duration of the hospital stay.

### Statistical analyses

Statistical analyses were performed using the Mann–Whitney U‐test and Fisher's exact test with EZR (Saitama Medical Center, Jichi Medical University), which is a graphical user interface for R (The R Foundation for Statistical Computing). More precisely, this is a customized version of R commander tailored to incorporate frequently used statistical functions in biostatistics. Statistical significance was defined as *p* < 0.05.

### Study organization and funding

This study was managed by the Department of Gastroenterology and Hepatology, Niigata University Medical and Dental Hospital, and funded by Daiichi Sankyo Co., Ltd. The sponsor had no role in the study design, conduct of the study, data collection and analysis, decision to publish, or preparation of the manuscript.

## RESULTS

### Patient characteristics

We performed 21 endoscopy procedures with a high risk of bleeding in 21 patients who were on edoxaban for anticoagulation. The characteristics of the patients are shown in Table 1. Three of the 21 patients were preoperatively switched from warfarin to edoxaban.

**TABLE 1:** Patient characteristics.

Patients, *n*	21
Age (years), median (range)	73 (58–83)
Sex, male/female	14/7
Alcohol, *n* (%)	8 (38)
Smoking, *n* (%)	4 (19)
Diabetes, *n* (%)	3 (14)
Hemodialysis, *n* (%)	0 (0)
Hypertension, *n* (%)	16 (76)
BMI (kg/m^2^), mean ± SD	22.9 ± 4.8
SBP (mmHg), mean ± SD	123.3 ± 19.1
Platelet (/μL), mean ± SD	24.0 ± 6.6
eGFR (mL/min), mean ± SD	62.8 ± 18.4
**Anticoagulant medication**	
Edoxaban (*n*)	18
Replacing warfarin with edoxaban (*n*)	3
Dose of Edoxaban, 60 mg/30 mg/15 mg	7/12/2
Hospital stay (days), median (range)	8 (3–24)

Abbreviations: BMI, body mass index; eGFR, estimated glomerular filtration rate; SBP, systolic blood pressure.

### Endoscopic procedures

The procedures performed in this study included 14 ESDs, five EMRs, one polypectomy, and one POEM. The treated organs were the esophagus in five cases, the stomach in eight cases, the duodenum in two cases, and the colorectum in six cases. The types of procedures and organs, as well as the number of resected lesions, are shown in Table 2.

**TABLE 2:** Procedures.

	Esophagus	Stomach	Duodenum	Colorectum
ESD	4	7^†^		3
EMR			2	3^‡^
Polypectomy		1		
POEM	1			

Abbreviations: EMR, endoscopic mucosal resection; ESD, endoscopic submucosal dissection; POEM, per‐oral endoscopic myotomy.

^†^ One case with two lesions resected and one case with four lesions resected were included.

^‡^ One case with two lesions resected and one case with three lesions resected were included.

### Delayed bleeding and other adverse events

**FIGURE 1:**
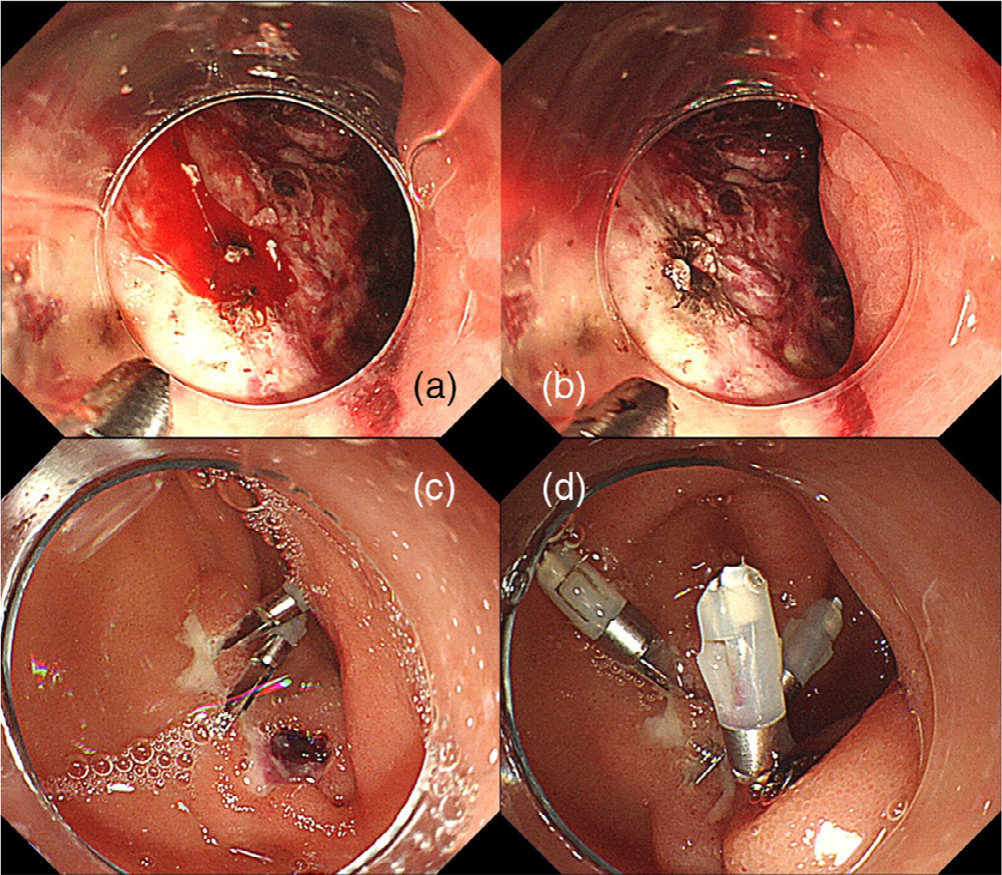
(a) Active bleeding occurred in post‐gastric endoscopic submucosal dissection (ESD) ulcer on the first postoperative day. (b) Post‐endoscopic submucosal dissection ulcer after hemostasis with coagulation forceps. (c) Blood vessel is observed in post‐duodenal endoscopic mucosal resection ulcer on the 6th postoperative day. (d) Hemostatic clip (HX‐610‐135; Olympus) was placed in the blood vessel.

CTCAE v5.0 Grade III–V delayed bleeding occurred in three of the 21 patients (14%), including two that had undergone gastric ESD and one that underwent duodenal EMR. Delayed bleeding occurred on postoperative days 1 and 3 in the gastric ESD cases and on postoperative day 6 in the duodenal EMR case. In the gastric ESD cases, coagulation hemostasis was performed using hemostatic forceps, whereas hemostatic clips were used in the duodenal EMR case (Figure 1). Endoscopic hemostasis was performed successfully in all cases, and the patients recovered with conservative management. There were no CTCAE v5.0 grade I–II delayed bleeding events. All delayed bleeding occurred during hospitalization, and no bleeding adverse events occurred during the 2‐month follow‐up period after discharge. No thromboembolic adverse events occurred in this study. Other adverse events included cholangitis and aspiration pneumonia; these patients recovered with conservative treatment.

### Length of hospital stay

The median length of hospital stay was 8 days (3–24) in this study.

### Comparison with and without delayed bleeding

Table 3 shows a comparison of procedure types, patient characteristics, and length of hospital stays between groups with and without delayed bleeding. In this study, significant differences were found in the systolic blood pressure at admission and the length of hospital stay.

**TABLE 3:** Characteristics of patients, procedures, and lesions compared with or without delayed bleeding

	Delayed bleeding	No delayed bleeding	
	(*n* = 3)	(*n* = 18)	*p*‐value
Age ≥70 years (Y/N), *n*	3/0	12/6	0.62
Sex (male/female), *n*	2/1	12/6	0.75
BMI ≥25 k/m^2^ (Y/N), *n*	3/0	13/5	0.42
SBP ≥140 mmHg (Y/N), *n*	2/1	1/17	**0.04**
Alcohol (Y/N), *n*	0/3	8/10	0.21
Smoking (Y/N), *n*	0/3	4/14	0.91
Diabetes (Y/N), *n*	1/2	2/16	0.89
Hypertension (Y/N), *n*	1/2	2/16	0.75
Platelet ≤ 15/μL (Y/N), *n*	0/3	1/17	0.85
eGFR ≤ 70 mL/min (Y/N), *n*	2/1	12/6	0.75
**Anticoagulant medication**			
Replacing warfarin with Edo (Y/N), *n*	1/2	2/16	0.95
Dose of Edo (60/30/15mg), *n*	1/1/1	6/11/1	0.30
**Procedures and lesions**			
Procedures (ESD/EMR/Pol/POEM), *n*	2/1/0/0	12/4/1/1	0.92
Location (Eso/Sto/Duo/Col), *n*	0/2/1/0	5/6/1/6	0.19
Resection of multiple lesions (Y/N), *n*	1/2	3/15	0.92
Lesion size ≥ 20 mm (Y/N)	3/0	6/11^†^	0.07
Hospital stay (days), median (range)	13 (10–24)	8(3–24)	**0.02**

Abbreviations: BMI, body mass index; Col, Colorectum; Duo, duodenum; Edo, Edoxaban; eGFR, estimated glomerular filtration rate; EMR, endoscopic mucosal resection; ESD, endoscopic submucosal dissection; Eso, Esophagus; Pol, polypectomy; SBP, systolic blood pressure; Sto, stomach.

^†^ The case of POEM was excluded.

## DISCUSSION

Considering clinical practice, the proportion of patients taking antithrombotic drugs is increasing, so it is important to consider the safety of endoscopic procedures in these patients. In particular, the development of novel anticoagulants, such as DOACs, has led to changes in the types of antithrombotic drugs being used.

In this study, we prospectively investigated the impact of administering the DOAC edoxaban during gastrointestinal endoscopic procedures with a high risk of bleeding. Most previous reports on the effects of DOACs in endoscopic procedures have focused on ESD and have been retrospective studies. Murata et al. have reported on the association between DOACs and delayed bleeding after ESD from the viewpoint of pharmacokinetics from a prospective study^26^; however, the number of prospective studies is still small. This study is one of the few prospective studies of its kind.

Bleeding is an important incident related to invasive procedures in patients taking antithrombotic drugs, but cardiovascular and cerebrovascular complications due to thrombosis are often serious and should be given the most attention. The most important point in interpreting the results is that no thrombosis‐related complications were observed in the perioperative period in this study.

As previously reported, appropriate anticoagulant interruption reduces the bleeding rate. However, it has also been noted that concurrent use of anticoagulants and antiplatelet agents may lead to an increase in delayed bleeding rates. Patients concurrently using antiplatelet agents were excluded from this study, with edoxaban alone being evaluated.

The overall incidence of delayed bleeding in this study was 14% (3/21). In an investigation of the incidence of delayed bleeding in high‐risk endoscopic procedures using the Japanese Diagnosis Procedure Combination database, the rate for DOACs was 9.9%, which was similar.^16^ Even when limited to ESD procedures, the rate was 14% (2/14). Previous studies focusing on ESD have reported rates from 13% to 22%, and the results of this study were within a similar range.^17^, ^18^, ^19^, ^20^, ^21^, ^22^, ^23^, ^24^, ^25^, ^26^ Among the previously reported data specific to edoxaban as a DOAC, the delayed bleeding rate was 9.5%.^18^ Additionally, a retrospective analysis of delayed bleeding following ESD under sole DOAC administration, without concurrent antiplatelet use, at our institution from August 2012 to August 2022, showed rates of 11.6% (5/43) for DOACs overall and 11.7% (2/17) for edoxaban alone, consistent with the results of this study. Although pharmacokinetic properties differ significantly among DOACs,^24^ this study could not detect any difference in the rate of delayed bleeding between edoxaban and other DOACs in endoscopic procedures with a high risk of bleeding.

In this study, systolic hypertension on admission was identified as a risk factor for delayed bleeding in endoscopic procedures under edoxaban administration; however, there was no association between a history of hypertension treatment and delayed bleeding. Li et al. reported that hypertension is a comorbidity that is a risk factor for delayed bleeding during colorectal ESD,^33^ but it has not been listed as a risk factor in various reports on patients taking DOACs.^17^, ^18^, ^26^ Although poor perioperative blood pressure control may be a risk factor for delayed bleeding, it is difficult to comment on the relationship because the blood pressure at admission in this study fluctuated according to the patient's condition. Previous reports have identified lesion size, age, sex, dialysis, and procedure duration as risk factors,^17^, ^18^, ^26^ but these were not identified in this study. Specific risk factors useful for predicting delayed bleeding during endoscopic procedures in patients taking edoxaban weren't identified in this study.

In reports limited to ESD, no differences in the risk of delayed bleeding based on the type of DOACs have been reported. However, some reports indicate that edoxaban has a lower incidence of bleeding events compared to other DOACs, suggesting that edoxaban may also contribute to reducing the risk of delayed bleeding associated with endoscopic procedures.

The median length of hospital stay in this study was 8 days, and the mode was also 8 days. This is thought to be because the length of hospital stays for the clinical pathway for ESD, which was the most common procedure, was 8 days. Yoshiya et al. reported that the average length of hospital stays for gastric ESD, combined with heparin replacement from warfarin, was 22.5 days, and for DOACs it was approximately 7 days; this study had a similar duration. In contrast, considering the observed extension of hospital stays in the post‐bleeding group, the management of delayed bleeding is important to reduce the length of the hospital stay.

The limitation of this study was that it was a single‐center study without a control group and a small number of cases.

The study originally planned to enroll 40 cases but was limited to 21 cases. The cause was thought to be that there were fewer patients taking warfarin than expected, that many patients were already taking DOACs other than edoxaban, and that more patients were taking concomitant antiplatelet drugs than expected. Considering clinical practice, the risk of delayed bleeding should be investigated, including that in patients receiving concomitant antiplatelet treatment.

Another limitation is the small number of procedures other than ESD. Nagata et al. reported that the rate of delayed bleeding after ESD in patients taking DOACs was higher than that after EMR.^16^ The number of procedures other than ESD should be increased to clarify the effect of different procedures on delayed bleeding in patients taking edoxaban.

Additionally, the small number of cases for each organ is a limitation when considering the effects of differences in organs. To clarify the influence of different organs on the delayed bleeding rate in patients taking edoxaban, the number of procedures for each organ should be increased.

In conclusion, the delayed bleeding incidence of endoscopic procedures with a high risk of bleeding in patients taking edoxaban with appropriate discontinuation was acceptable in this prospective study. Hypertension may be a risk factor for delayed bleeding, but to clarify this, it is necessary to consider factors such as medication history and postoperative blood pressure management. In addition, to clarify the effect of different types of procedures and organs on delayed bleeding in patients taking edoxaban, further research on procedures other than ESD and of each organ is necessary.

## CONFLICT OF INTEREST STATEMENT

Ken‐ichi Mizuno received honoraria from Daiichi‐Sankyo, Takeda Pharmaceutical, Olympus, Fujifilm, Miyarisan Pharmaceutical, and Nipro. Shuji Terai received research grants from Interstem, ASKA Pharmaceutical, Tsumura, CHIOME Bioscience, BioMimetics Sympathies, Nihon Pharmaceutical, Sysmex, Tosoh, Rohto, Stemrim, Shionogi, Kowa, Gilead Sciences, Abbott, AbbVie, Sumitomo Pharma, Takeda Pharmaceutical, Nippon Kayaku, Otsuka Pharmaceutical, Eisai, EA Pharma, and Asahi Kasei Pharma, and honoraria from Diichi‐Sankyo, Takeda Pharmaceutical, ASKA Pharmaceutical, Gilead Sciences, MSD, and Otsuka Pharmaceutical. The other authors declare no conflict of interest.

## ETHICS STATEMENT

The study was approved by the Institutional Review Board of the Niigata University Medical and Dental Hospital (approval no. 2016‐0054). The study was performed in accordance with the Declaration of Helsinki and Good Clinical Practice guidelines.

## PATIENT CONSENT STATEMENT

Written informed consent was obtained from all the patients in the study.

## CLNICAL TRIAL REGISTRATION

The study protocol was registered in the Japan Registry of Clinical Trials (jRCT; jRCTs031180190).

## Data Availability

The data that support the findings of this study are available from the corresponding author, K.M., upon reasonable request.

## References

[1] Jørgensen HS , Nakayama H , Reith J , Raaschou HO , Olsen TS . Acute stroke with atrial fibrillation. The Copenhagen stroke study. Stroke 1996; 27: 1765–1769. 8841326 10.1161/01.str.27.10.1765

[2] Lansberg MG , O'Donnell MJ , Khatri P *et al.* Antithrombotic and thrombolytic therapy for ischemic stroke: Antithrombotic therapy and prevention of thrombosis, 9th ed: American College of Chest Physicians evidence‐based clinical practice guidelines. Chest 2012; 141: e601S–e636S. 22315273 10.1378/chest.11-2302 PMC3278065

[3] Wolf PA , Abbott RD , Kannel WB . Atrial fibrillation as an independent risk factor for stroke: The Framingham study. Stroke 1991; 22: 983–988. 1866765 10.1161/01.str.22.8.983

[4] Okada K , Yamamoto Y , Kasuga A *et al.* Risk factors for delayed bleeding after endoscopic submucosal dissection for gastric neoplasm. Surg Endosc 2011; 25: 98–107. 20549245 10.1007/s00464-010-1137-4

[5] Takizawa K , Oda I , Gotoda T *et al.* Routine coagulation of visible vessels may prevent delayed bleeding after endoscopic submucosal dissection–An analysis of risk factors. Endoscopy 2008; 40: 179–183. 18322872 10.1055/s-2007-995530

[6] Akasaka T , Nishida T , Tsutsui S *et al.* Short‐term outcomes of endoscopic submucosal dissection (ESD) for early gastric neoplasm: Multicenter survey by Osaka University ESD study group. Dig Endosc 2011; 23: 73–77. 21198921 10.1111/j.1443-1661.2010.01062.x

[7] Takeuchi T , Ota K , Harada S *et al.* The postoperative bleeding rate and its risk factors in patients on antithrombotic therapy who undergo gastric endoscopic submucosal dissection. BMC Gastroenterol 2013; 13: 136. 24010587 10.1186/1471-230X-13-136 PMC3844538

[8] Koh R , Hirasawa K , Yahara S *et al.* Antithrombotic drugs are risk factors for delayed postoperative bleeding after endoscopic submucosal dissection for gastric neoplasms. Gastrointest Endosc 2013; 78: 476–483. 23622974 10.1016/j.gie.2013.03.008

[9] Yoshio T , Nishida T , Kawai N *et al.* Gastric ESD under heparin replacement at high‐risk patients of thromboembolism is technically feasible but has a high risk of delayed bleeding: Osaka University ESD study group. Gastroenterol Res Pract 2013; 2013: 365830. 23843783 10.1155/2013/365830 PMC3697307

[10] Ono S , Fujishiro M , Niimi K *et al.* Technical feasibility of endoscopic submucosal dissection for early gastric cancer in patients taking anti‐coagulants or anti‐platelet agents. Dig Liver Dis 2009; 41: 725–728. 19230799 10.1016/j.dld.2009.01.007

[11] Yoshio T , Tomida H , Iwasaki R *et al.* Effect of direct oral anticoagulants on the risk of delayed bleeding after gastric endoscopic submucosal dissection. Dig Endosc 2017; 29: 686–694. 28295638 10.1111/den.12859

[12] Connolly SJ , Ezekowitz MD , Yusuf S *et al.* Dabigatran versus warfarin in patients with atrial fibrillation. N Engl J Med 2009; 361: 1139–1151. 19717844 10.1056/NEJMoa0905561

[13] Granger CB , Alexander JH , McMurray JJ *et al.* Apixaban versus warfarin in patients with atrial fibrillation. N Engl J Med 2011; 365: 981–992. 21870978 10.1056/NEJMoa1107039

[14] Patel MR , Mahaffey KW , Garg J *et al.* Rivaroxaban versus warfarin in nonvalvular atrial fibrillation. N Engl J Med 2011; 365: 883–891. 21830957 10.1056/NEJMoa1009638

[15] Giugliano RP , Ruff CT , Braunwald E *et al.* Edoxaban versus warfarin in patients with atrial fibrillation. N Engl J Med 2013; 369: 2093–2104. 24251359 10.1056/NEJMoa1310907

[16] Nagata N , Yasunaga H , Matsui H *et al.* Therapeutic endoscopy‐related GI bleeding and thromboembolic events in patients using warfarin or direct oral anticoagulants: Results from a large nationwide database analysis. Gut 2018; 67: 1805–1812. 28874418 10.1136/gutjnl-2017-313999 PMC6145295

[17] Sanomura Y , Oka S , Tanaka S *et al.* Taking warfarin with heparin replacement and direct oral anticoagulant is a risk factor for bleeding after endoscopic submucosal dissection for early gastric cancer. Digestion 2018; 97: 240–249. 29421806 10.1159/000485026

[18] Tomida H , Yoshio T , Igarashi K *et al.* Influence of anticoagulants on the risk of delayed bleeding after gastric endoscopic submucosal dissection: A multicenter retrospective study. Gastric Cancer 2021; 24: 179–189. 32683602 10.1007/s10120-020-01105-0

[19] Horie Y , Horiuchi Y , Ishiyama A *et al.* The effect of antithrombotic drug use on delayed bleeding with esophageal endoscopic resection. J Gastroenterol Hepatol 2022; 37: 1792–1800. 35844140 10.1111/jgh.15944

[20] Saito H , Igarashi K , Hirasawa D *et al.* The risks and characteristics of the delayed bleeding after endoscopic submucosal dissection for early gastric carcinoma in cases with anticoagulants. Scand J Gastroenterol 2020; 55: 1253–1260. 32924673 10.1080/00365521.2020.1817542

[21] Yamashita K , Oka S , Tanaka S *et al.* Use of anticoagulants increases risk of bleeding after colorectal endoscopic submucosal dissection. Endosc Int Open 2018; 6: E857–E864. 29978006 10.1055/a-0593-5788 PMC6031438

[22] Harada H , Nakahara R , Murakami D *et al.* The effect of anticoagulants on delayed bleeding after colorectal endoscopic submucosal dissection. Surg Endosc 2020; 34: 3330–3337. 31482349 10.1007/s00464-019-07101-5

[23] Li R , Cai S , Sun D *et al.* Risk factors for delayed bleeding after endoscopic submucosal dissection of colorectal tumors. Surg Endosc 2021; 35: 6583–6590. 33237467 10.1007/s00464-020-08156-5

[24] Sugimoto M , Murata M , Kawai T . Assessment of delayed bleeding after endoscopic submucosal dissection of early‐stage gastrointestinal tumors in patients receiving direct oral anticoagulants. World J Gastroenterol 2023; 29: 2916–2931. 37274799 10.3748/wjg.v29.i19.2916 PMC10237096

[25] Higuchi K , Goto O , Matsuda A *et al.* Potential of direct oral anticoagulant in bleeding after gastric endoscopic submucosal dissection: A systematic review and meta‐analysis. Dig Dis Sci 2024; 69: 940–948. 38252209 10.1007/s10620-024-08271-6

[26] Murata M , Sugimoto M , Ueshima S *et al.* Association of direct oral anticoagulant and delayed bleeding with pharmacokinetics after endoscopic submucosal dissection. Gastrointest Endosc 2024; 99: 721–731.e4. 38042206 10.1016/j.gie.2023.11.048

[27] Sugimoto M , Hatta W , Tsuji Y *et al.* Risk factors for bleeding after endoscopic submucosal dissection for gastric cancer in elderly patients older than 80 years in Japan. Clin Transl Gastroenterol 2021; 12: e00404. 34644281 10.14309/ctg.0000000000000404 PMC8659993

[28] Kato ET , Giugliano RP , Ruff CT *et al*. Efficacy and safety of edoxaban in elderly patients with atrial fibrillation in the engage AF‐TIMI 48 trial. J Am Heart Assoc 2016; 5: e003432. 27207971 10.1161/JAHA.116.003432 PMC4889207

[29] Xu W , Lv M , Wu S *et al.* Severe bleeding risk of direct oral anticoagulants versus vitamin K antagonists for stroke prevention and treatment in patients with atrial fibrillation: A systematic review and network meta‐analysis. Cardiovasc Drugs Ther 2023; 37: 363–377. 34436708 10.1007/s10557-021-07232-9

[30] Inoue T , Nishida T , Maekawa A *et al.* Clinical features of post‐polypectomy bleeding associated with heparin bridge therapy. Dig Endosc 2014; 26: 243–249. 23730922 10.1111/den.12123

[31] Kato M , Uedo N , Hokimoto S *et al.* Guidelines for gastroenterological endoscopy in patients undergoing antithrombotic treatment: 2017 appendix on anticoagulants including direct oral anticoagulants. Dig Endosc 2018; 30: 433–440. 29733468 10.1111/den.13184

[32] Fujimoto K , Fujishiro M , Kato M *et al.* Guidelines for gastroenterological endoscopy in patients undergoing antithrombotic treatment. Dig Endosc 2014; 26: 1–14. 10.1111/den.12183 24215155

[33] Li R , Cai S , Sun D . Risk factors for delayed bleeding after endoscopic submucosal dissection of colorectal tumors. Surg Endosc 2021; 35: 6583–6590. 33237467 10.1007/s00464-020-08156-5

